# Supplementary material for: A socio-ecological examination of the primary school playground: Primary school pupil and staff perceived barriers and facilitators to a physically active playground during break and lunch-times
Source: PLoS One. 2022 Feb 2;17(2):e0261812. doi: 10.1371/journal.pone.0261812 (PMC8809583; doi:10.1371/journal.pone.0261812)
Supplement: S1 File — (DOCX) [file pone.0261812.s001.docx]

**Supplementary information**

**S1. Focus group and interview topic guide**

**Barriers and facilitators to a physically active playground**

Please use the topics below when facilitating discussions around barriers and facilitators

If staff members prefer they can fill out their opinions on the topics below. Please use additional sheets of paper where necessary as the space provided below may be insufficient.

1. – What things prevent or promote physical activity during break-times? Why? Does anything need to change?

*For children, use the map of the playground and write the things about each area that help or prevent you from taking part in PA – Playground as a whole and then each individual area*

2. – What skills (physical, social and mental) do you think children need to be active during break-times? (for example, if equipment is provided – what prerequisite skills do children need to use the equipment effectively?

*For children - New map – write down all the skill requirements you think are needed in each of the areas of your playground (provide post-its if needed)*

3. – What do you think the role of the playground supervision is? How are they perceived by the pupils and what do you think the effects of their presence has on physical activity levels?

*For children - Draw your idea of a supervisor – write some words about what their responsibilities are? What do you think of when you are drawing them?*

4. - Should any future playground intervention/activities be research led, teacher led, pupil/peer led?). Please explain your answer (facilitators to encourage discussion)

5. - Should future intervention/activities be designed to challenge individual children or designed for the playground population as a whole

6. **Magic wish** - If you (the staff member) could request one thing for the children to use at break-time, what would it be?  Please could you expand on your answer.

*For children – provide children with a sheet of paper/post-it. Ask the child to write their magic wish, in secret and post it in the box.*
