# Supplementary material for: A socio-ecological examination of the primary school playground: Primary school pupil and staff perceived barriers and facilitators to a physically active playground during break and lunch-times
Source: PLoS One. 2022 Feb 2;17(2):e0261812. doi: 10.1371/journal.pone.0261812 (PMC8809583; doi:10.1371/journal.pone.0261812)
Supplement: Attachment — Submitted filename: Review - PONE-D-21-28206.pdf [file pone.0261812.s002.pdf]

Review - Plos One - PONE-D-21-28206

General comments: First congratulate the authors for the research, in my view the theme is extremely important and it takes empathy to transcend the findings, I judge the relevant research and I am grateful to be able to review this manuscript. I have small comments on points of the manuscript that in my view should be improved. They are:

Abstract

- In this session the authors state "Using the socio-ecological model", however do not characterize such a model, I suggest informing which model the socio-ecological was used.
- The authors mention the total number of children and the chronological age margin, however, how many adults participated in the study? What are your school roles? Teachers? And what is the chronological age margin of adults?
- What were the differences perceived by children and adults?
- What are the environmental limits and factors of school policy that are interrelated?
- The authors inform what the study offers, that is, a perspective of practical applicability. However, the abstract does not point to a conclusion. What the study concludes based on the findings?
- Authors need to insert at least three keywords after the abstract.

Introduction

- Line 104 – Need a reference to end of sentence.
- Line 111 – In the introduction prioritize the passive voice, leave the active voice for the discussion session.
- Final paragraph of the introduction: The authors need to present a hypothesis after mentioning the objective, which they believed before the study began?

Methods

- The authors need to insert in the session called "Participants", the specific functions of each adult who was part of the sample, for example how many were teachers? You need to provide more details in this session such as the age group of adults for example.

Results

- No comments.

Discussion

- The additional results should be in the results session and not in the discussion session. The authors should present all the findings in the results session. Later, in the discussion session should start informing the objective of the study, the initial hypothesis and inform through which results the initial hypothesis was

proven (or not). Thus, a discussion should be held with the existing data in the literature.

Summary and conclusion

- Set the title of this session only to “Conclusions”.
- The conclusion should answer the objective of the work. All the rest (limitations, observations, views, suggestions, future prospects) should come to the end of the discussion session.
- This session is very extensive, sift the conclusion of the study to a maximum of ten lines in a single paragraph.
