# Supplementary material for: A socio-ecological examination of the primary school playground: Primary school pupil and staff perceived barriers and facilitators to a physically active playground during break and lunch-times
Source: PLoS One. 2022 Feb 2;17(2):e0261812. doi: 10.1371/journal.pone.0261812 (PMC8809583; doi:10.1371/journal.pone.0261812)
Supplement: Attachment — Submitted filename: Author-response_V1.docx [file pone.0261812.s004.docx]

Manuscript title: ' A socio-ecological examination of the primary school playground: primary school pupil and staff perceived barriers and facilitators to a physically active playground during break and lunch-times '

We would like to thank the editor and reviewers for their comments and recommendations. We have considered all the suggestions and have provided our point-by-point response below which is cross referenced by line number to the version of the manuscript including tracked changes (‘revised manuscript with tracked changes’). We also provide an amended version of the manuscript with changes accepted (‘manuscript’). All references, in text and in the reference list, have been updated accordingly. We feel the recommendations have contributed to an improved overall manuscript.

| **Comments** | **Authors’ response** |
| --- | --- |
| Reviewer #1  First congratulate the authors for the research, in my view the theme is extremely important and it takes empathy to transcend the findings, I judge the relevant research and I am grateful to be able to review this manuscript. I have small comments on points of the manuscript that in my view should be improved. | We would like to thank reviewer one for their feedback and kind words. We have attempted to address each of the comments below. |
| **Abstract**  In this session the authors state "Using the socio-ecological model", however do not characterize such a model, I suggest informing which model the socio-ecological was used. | On the recommendation of reviewer one, we have added a reference to Brofenbrenner’s model (line 27) and added the factors which characterize the model (line29-30). The details of this model and how it has been applied are presented in detail throughout the manuscript. |
| The authors mention the total number of children and the chronological age margin, however, how many adults participated in the study? What are your school roles? Teachers? And what is the chronological age margin of adults? | The number of children and staff members taking part in the study has now been added to the abstract at line 30-31. This detail is also available in the methods section.  The age range for the children is provided to inform the international audience of the age ranges of these UK school age groups. We did not collect the age ranges of the staff participating in the study, nor did we collect their role within the school. Whilst there was an option for this on the questionnaire – the staff members wished to maintain their anonymity and opted to withhold this information. This information can be found in the manuscript at line 545. |
| What were the differences perceived by children and adults? | We have considered including some of the differences between children and adults here. However, to select one (or a couple) as an example would lead us to ‘select’ factors by our perceived importance. Therefore, we have included a sentence to highlight that child/adult differences were observed, at line 38-40. |
| What are the environmental limits and factors of school policy that are interrelated? | Please see point above regarding selecting examples. However, Line 35-36 highlights that these barriers centre around resource availability, accessibility, and health and safety. |
| The authors inform what the study offers, that is, a perspective of practical applicability. However, the abstract does not point to a conclusion. What the study concludes based on the findings? | A sentence has been added at line 39-40, highlighting how break-times are affected by a number of variables at each level of the socio-ecological model. |
| Authors need to insert at least three keywords after the abstract. | We acknowledge this request and would be happy to provide some keywords. However, this is not a requirement according to the submission/author guidelines provided by PLOS ONE. |
| **Introduction**  Line 104 – Need a reference to end of sentence | Reference number 28; *The Youth sport trust (2018) How to spend it: A guide to Primary PE and Sport Premium*, has been added at line 106. |
| Line 111 – In the introduction prioritize the passive voice, leave the active voice for the discussion session | This passage has been amended to reflect a more passive writing style. Line 113-116 |
| Final paragraph of the introduction: The authors need to present a hypothesis after mentioning the objective, which they believed before the study began? | Whilst we understand the reviewers comment here, we would disagree that a hypothesis is needed in the current manuscript. On review of other PLOS ONE qualitative articles there seems to be no consensus on the inclusion or not of a ‘traditional’ hypothesis. In this case we do not think it would add value. |
| **Methods**  The authors need to insert in the session called "Participants", the specific functions of each adult who was part of the sample, for example how many were teachers? You need to provide more details in this session such as the age group of adults for example | Whilst we agree that identifying the staff roles would give an additional line of discussion (i.e., behaviour differences between those supervising) we were unable to collect this information (see line 268-277 in methods and 545 in discussion).  We include in Table 2, the number of staff members. We include the roles within the school that we targeted/included (line 173), however we cannot identify the roles of the 11 staff members. |
| **Discussion**  The additional results should be in the results session and not in the discussion session. The authors should present all the findings in the results session. Later, in the discussion session should start informing the objective of the study, the initial hypothesis and inform through which results the initial hypothesis was proven (or not). Thus, a discussion should be held with the existing data in the literature. | We would like to thank reviewer one for their thoughts on this point. Whilst we see the value of separating the results and discussion in most cases, in the current manuscript we believe it would weaken the critical discussion we present. We have added a section at the start of the discussion (Line 352) to acknowledge that whilst this might be a break from ‘tradition’ the authors believe the interwoven discussion with real world accounts from the data is a real strength of this manuscript. Further, by extracting the data (transcripts) from the discussion and placing it in the results would cause the reader to have to refer back and forwards to the referenced transcripts, breaking up the flow for the reader. |
| **Summary and Conclusion**  Set the title of this session only to “Conclusions” | This section has been re-formatted to include implications for practice (section 5 line 596), strengths and weaknesses (section 6 line 639) and a separate conclusion (section 7 line 667). |
| The conclusion should answer the objective of the work. All the rest (limitations, observations, views, suggestions, future prospects) should come to the end of the discussion session. | See comment above – the conclusion has been separated from the other sections |
| This session is very extensive, sift the conclusion of the study to a maximum of ten lines in a single paragraph | The inclusion of a separate conclusion (section 7 line 667) has made it easier to provide a more concise conclusion (12 lines). |
| Reviewer #2  The authors present a document that is ethically and academically acceptable. They use the robust theoretical framework with some expertise. Even so, I suggest that the research question and the hypotheses allied to the Theory used be presented more clearly. In certain passages the paragraphs are quite long which can interfere with the fluidity of the reading | Line 89, 100, 203, 293, 650 paragraphs have been split to address reviewer two’s comment here. In addition sections in the discussion have been split/shortened in response to reviewer one’s request. |
| Journal requirements:  Please ensure that your manuscript meets PLOS ONE's style requirements, including those for file naming. | Level 1, 2 and 3 titles have been amended to 18, 16 and 14 point text, respectively.  Figures have been amended to read ‘Fig 1, Fig 2’ etc. in the text and In the figure titles.  Supporting material has been added and named according to the style guidelines. |
| Please include additional information regarding the survey, questionnaire, or interview guides used in the study and ensure that you have provided sufficient details that others could replicate the analyses | The topic guide for the focus groups and interviews is included in S1 Supporting information_Focus group and interview topic guide. |
| In your Data Availability statement, you have not specified where the minimal data set underlying the results described in your manuscript can be found. | We had originally ticked the *‘all data are fully available without restriction’* however, on reflection some of the transcriptions and pictures have person/school identifiable information on and therefore can’t be shared. We have changed the statement to now read *‘No-some restrictions will apply’*. However, we believe the manuscript includes sufficient examples of these data collection outputs. |
| Please review your reference list to ensure that it is complete and correct. If you have cited papers that have been retracted, please include the rationale for doing so in the manuscript text, or remove these references and replace them with relevant current references. Any changes to the reference list should be mentioned in the rebuttal letter that accompanies your revised manuscript. If you need to cite a retracted article, indicate the article’s retracted status in the References list and also include a citation and full reference for the retraction notice | Reference number 69 (line 907) - Martínez-Andrés M et al. 2017, has been amended to include the reference to the correct volume and issue number. |
